# Reply to the Editor— Functional substrate mapping of ventricular arrhythmia

**DOI:** 10.1016/j.hroo.2024.11.017

**Published:** 2024-11-22

**Authors:** Konstantinos Vlachos, Antonio Frontera, Karim Benali, Konstantinos P. Letsas, Pierre Jais, Pasquale Santangeli

**Affiliations:** 1 IHU LIRYC ANR-10-IAHU-04, Centre Hospitalier Universitaire Bordeaux, Bordeaux, France; 2 Electrophysiology Unit, De Gasperis Cardio Center, Niguarda Hospital, Milan, Italy; 3 Electrophysiology Department, Onassis Cardiac Surgery Center, Athens, Greece; 4 Section of Cardiac Pacing and Electrophysiology, Department of Cardiovascular Medicine, Cleveland Clinic, Cleveland, Ohio

Outcomes from ventricular tachycardia (VT) ablation vary significantly, with an average freedom from appropriate implantable cardioverter-defibrillator therapy of 72% for ablation compared with 60% for medical therapy in major randomized trials.^1^^,^^2^ These findings underscore the limitations of current ablation strategies, which fall short of optimal success rates that might improve nonarrhythmic outcomes such as mortality. However, VT ablation has demonstrated benefits, including reduced hospitalization, improved quality of life, and greater cost-effectiveness. It is important to note that real-world outcomes may be worse than those observed in controlled trial settings, emphasizing the need for advancements in ablation techniques. In this context, our review focuses on functional substrate mapping approaches to improve the identification of abnormal VT substrates in patients with structural heart disease.

We appreciate the thoughtful comments from Drs Hsia and Choi and agree that further investigations are needed to determine the optimal activation wavefront for substrate mapping, which varies depending on substrate location. For example, Shinoda et al^3^ elegantly demonstrated that rotational activation patterns (RAPs) around localized conduction blocks are often associated with critical VT zones. The direction of the activation wavefront significantly affects the manifestation of RAP and the location of lines of block (LOBs). Pacing near the scar can enhance identification of critical VT zones, but the optimal rhythm for substrate mapping depends on the spatial distribution of the target substrate.^3^ RAPs and LOBs are more apparent when the wavefront is perpendicular to the LOBs, particularly when delayed conduction is present on one side. However, when multiple wavefronts collide late in the activation process, identifying RAPs and LOBs becomes challenging.

Practically, the activation wavefront chosen for substrate mapping is often based on the preprocedural imaging distribution of the abnormal substrate. For anterior left ventricular (LV) scars, sinus rhythm or right ventricular (RV) pacing is preferred; for septal LV scars, RV basal pacing is used; and for lateral LV scars, LV pacing from a coronary sinus branch is optimal. However, substrates involving both septal and lateral components may require multiple wavefronts to comprehensively identify slow conduction and block areas. Studies demonstrated that pacing from RV and LV sites can reveal an additional 25% of critical VT sites not evident during sinus rhythm mapping.^4^^,^^5^ Moreover, Nishimura et al^6^ showed that differential pacing can uncover concealed LOBs in 84% of regions without overt LOBs during baseline rhythms, highlighting the importance of pacing in regions of slow conduction.

Preemptive identification of isthmus boundaries during sinus or paced rhythms could simplify VT ablation, reduce the need for VT induction, and enhance procedural safety. In addition, accurate electrogram annotation is critical to mapping. Advances in automated annotation algorithms, such as optimization decremented evoked potential mapping annotation, could further refine substrate mapping.

Future developments may include integrating imaging modalities such as magnetic resonance imaging or computed tomography to identify arrhythmogenic substrates preprocedurally. These tools can guide substrate mapping, particularly in areas of scar tissue or lipomatous metaplasia, which are known to support VT reentry circuits. Another promising avenue for investigation is the role of repolarization heterogeneity in VT maintenance. For instance, Callans and Donahue^7^ found shorter activation-recovery intervals within the VT circuit isthmus compared with other regions, suggesting that disparities in repolarization may be key to sustaining VT. Investigating both depolarization abnormalities and repolarization heterogeneity could open new therapeutic avenues.

Finally, we thank Drs Hsia and Choi for their constructive feedback regarding Figures 1 and 3. A corrigendum will be issued in line with their suggestions to improve clarity and accuracy. We sincerely apologize for any oversights in the initial submission and are grateful for their contributions to refining our manuscript.
